# BO-LSTM-Based TDE Precise Estimation Model of Capacitive MEMS-Gyros Using Thermal-Induced Physical Characteristics Variation Analysis

**DOI:** 10.3390/mi17050508

**Published:** 2026-04-22

**Authors:** Bing Qi, Peng Li, Jicheng Ding, Chun Jia, Hao Tian

**Affiliations:** College of Intelligent Systems Science and Engineering, Harbin Engineering University, Harbin 150001, China; 18242096358@163.com (P.L.); dingjicheng@hrbeu.edu.cn (J.D.); jiachun@hrbeu.edu.cn (C.J.); tianzixvan@foxmail.com (H.T.)

**Keywords:** CMG, temperature dependence, thermal-induced physical characteristics variation analysis, complete TCQ traceability, BO-LSTM

## Abstract

Owing to the fact that the conventional Temperature Drift Errors (TDEs) precise estimation model of Capacitive MEMS-Gyros (CMG) has incomplete Temperature Correlated Quantities (TCQ) and an imperfect parameter identification method to reduce bias stability, a BO-LSTM-based TDE precise estimation model using thermal-induced physical characteristics variation analysis is proposed. By analyzing microstructural deformation in CMG- and Si-based materials’ stiffness deterioration caused by thermal-induced physical characteristics variation, complete TCQ are traced, including ambient temperature variation ∆*T* and its square root ∆*T*^1/2^ plus its higher orders (∆*T*^2^, ∆*T*^3^, ∆*T*^4^), a modified TDE precise estimation model is formed. Long Short-Term Memory (LSTM) is applied to identify the modified model’s parameters owing to the typical time series characteristics of TDE and TCQ. In addition, Bayesian Optimization (BO) is introduced in LSTM to show a good guide for LSTM’s optimal hyperparameters. The modified model is implemented with BO-LSTM and compared with the conventional model based on Radial Basis Function Neural Network (RBFNN) in bias stability. The experimental results show that the modified model can more accurately estimate the TDE of CMG in a timely and improves its bias stability by 20%, which decouples the temperature dependence of Si-based material significantly and enhances the environmental adaptability of CMG in complex conditions remarkably.

## 1. Introduction

Capacitive MEMS-Gyros have some merits like excellent miniaturization, low power consumption and small cost, and they are used in micro-inertial navigation for UAV or UUV, guidance, attitude detection and so on [1]. Owing to the fact that they are made up from Si-based material of temperature dependence, diverse ambient temperatures excite TDE to reduce the bias stability of CMG and restrict their application [2]. Due to limitations of current material technology, it is so hard to decouple temperature dependence by optimizing the production process. Temperature control of high accuracy and good stability can stabilize ambient temperature, but its high-power consumption, large volume, and complex control are inappropriate for CMG’s application [3]. With the merits of low cost and easy implementation, mathematical estimation uses a mathematical model to estimate precise TDE and compensate CMG in calculation, which is more proper for CMG [4]. Its accuracy depends on precise TDE traceability and accurate parameter identification of the TDE estimation model [5].

Precise TDE traceability is the foundation for the TDE estimation model [6]. More precise TDE traceability can figure out the root cause of TDE more comprehensively. Considering that the working principle of capacitive MEMS devices is invariant, universally, TCQ exciting TDE are consistent. By means of the experiments, Maj et al. conclude that ambient temperature variation of 1 °C causes a sensitivity variation 1% of the scale factor of the capacitive MEMS accelerometer, which reveals that varying ambient temperature is the direct cause of TDE [7]. Vatanparvar et al. show bias stability of MEMS gyro is influenced by a time-varying multi-physical coupling field, like incorporating temperature, angular rate and acceleration [8]. Bias and scale factor are expressed in temperature-dependent polynomials, and the regression method is used to obtain their coefficients [9,10,11,12]. Ma et al. researched a virtual temperature measurement whose measuring instability is 1.2 m°C based on the drive frequency immune to 1/f noise. They use it as the input of the TDE estimation model based on first-order polynomial fitting and angular velocity as its output [13]. By virtual measuring to the four-position Maytagging procedure, azimuth accuracy is increased from 0.47° to 0.26° and 0.35° within 5 min. Kim et al. show Si-based material deforms linearly as ambient temperature varies in a 3D structure [14]. A TDE estimation model is built with ambient temperature and TDE, and the bias stability of capacitive MEMS devices is enhanced by 10%. Bekkeng explores the relationship of TCQ and ambient temperature plus its variation’s square, and a Kalman filter is applied to estimate TDE with them [15]. The absolute rate error of MEMS inertial devices is reduced by more than a factor of 10. Shi et al. show that the thermal expansion property is nonlinear from a global perspective, and the deviations are 1~2 mg/°C in simulations and experiments [16]. Ambient temperature and its square plus ambient temperature variation and its square are TCQ, and a novel TDE estimation model is built with Back Propagation Neural Network (BPNN) using particle swarm optimization plus genetic algorithm, then bias stability of CMG is improved by 16.01% compared to the one using BPNN. Liu et al. and Wu et al. show that thermal expansion induced by ambient temperature variation causes CMG’s microstructural variation, and also deteriorates Si-based material’s stiffness to change the resonance frequency of its resonator and reduce bias instability [17,18]. Li et al. reveal that the resonance frequency deviation of two gyroscopic modes causes the measuring errors, which turns out that the root cause is stiffness deterioration caused by ambient temperature variation [19]. A frequency deviation calibration is implemented and bias repeatability of mode-matched MEMS gyro reaches 3.14°/h under 1*σ*, an improvement of three orders of magnitude compared to its original performance. So, the influence of ambient temperature on the resonance frequency variation in CMG is non-negligible. Thus, the key factor to precise TDE traceability is complete TCQ caused by microstructural deformation of CMG- and Si-based materials’ stiffness deterioration.

Accurate parameter identification is the requirement for the TDE estimation model [20]. More accurate parameters can ensure a more precise TDE is estimated. Cheng et al. introduce the particle swarm optimization algorithm to modify support vector machine models, and small batch data processing methods are used to guarantee its modeling real-time and accuracy [21]. It improves the bias stability of capacitive MEMS accelerometers to 18.96%, but support vector machine models with a complex structure are inappropriate to process abundant experimental results, and their real-time estimation has to be reduced. Li et al. establish a CMG temperature error model with a signal extraction method based on quantized temperature, and noise suppression is done by a Kalman filter and statistical calibration filter with an adaptive sliding window [22]. Bias instability is reduced to 1.10°/h in −40 °C~60 °C. Pan et al. show a TDE estimation model using a wavelet neural network, and bias stability after compensation is reduced to 10% of that before [23]. Ma et al. studied an IPSO algorithm for optimal VMD parameters to achieve the best decomposition denoising effect [24]. BP-Adaboost reconstructs the filtered mixed and compensated feature terms, which reduces the bias stability from 0.1806°/h to 7.17 × 10^−4^°/h. Xu et al. propose a TDE estimation model based on BPNN, and its nonlinear maximum is reduced from 3329 ppm to 603 ppm [25]. BPNN has a simpler structure, including an input layer and multiple hidden layers plus an output layer. It has excellent estimation performance to ensure higher precision, better real-time and easier implementation [26]. But, its local minima are prone to occur and bring non-optimal estimation when trained. To avoid that, Wang et al. train BPNN with a genetic algorithm [27]. After compensation, the maximum error of bias stability of MEMS accelerometers is 0.017% in −10~80 °C, 173-times better than the polynomial fitting method. The genetic algorithm has a probability disorder to reduce calculation accuracy and real-time performance, which increases training volume and parameter identification difficulty. RBFNN uses a function approximation method to describe the target nonlinearity as accurately as possible, and its three neural layers save calculation time to ensure estimation in real-time, even fast rotation [28]. Cheng et al. show a modified RBFNN-based temperature compensation model for IFOG, and it improves bias stability by more than an order of magnitude [29]. Li et al. show an improved Empirical Modal Decomposition (EMD) method and establish an EMD-based RBFNN+GA+KF fusion algorithm to compensate TDE [30]. Bias stability is reduced from 34.66°/h to 3.589°/h. Owing to the ambient temperature’s great inertia link, the bigger its variation is, the more time it takes to stabilize. So, ambient temperature is a time series, and mathematical models of time series characteristics are needed. Wang et al. construct a particle swarm optimization-support vector machine model to acquire temperature error by mid-frequency temperature signal [31]. An improved variational modal decomposition-extreme learning machine is proposed to separate CMG’s output signal, and is reconstructed with LSTM, which is a time series. The bias instability is reduced from 0.0087 to 1.8772 × 10^−4^ in −40~60 °C and 0.0145 to 7.2426 × 10^−4^ in 60~−40 °C. LSTM needs proper hyperparameters to ensure its performance, which requires adequate tuning. But, it takes much time, inordinate resources and many tries. A good guide enhances the search performance of the optimal hyperparameters. Thus, the key factor to accurate parameter identification lies in a good strategy to guide and obtain LSTM’s optimal hyperparameters.

In the paper, TDE traceability for CMG is explored using thermal-induced physical characteristics variation analysis, and complete TCQs are traced by analyzing microstructural deformation in CMG- and Si-based materials’ stiffness deterioration, including ∆*T*, ∆*T*^1/2^, ∆*T*^2^, ∆*T*^3^, ∆*T*^4^, and a modified TDE precise estimation model is established. LSTM is used in its parameter identification owing to typical time series characteristics of TDE and TCQ, and BO is introduced to offer a good guide for LSTM’s optimal hyperparameters; then, the modified model is implemented by BO-LSTM. The modified model can estimate TDE more precisely to decouple the temperature dependence of Si-based materials and enhance the environmental adaptability of CMG in complicated conditions.

## 2. Methodology

### 2.1. Modification of TDE Precise Estimation Model of CMG

#### 2.1.1. Conventional TDE Precise Estimation Model

**Figure 1:**
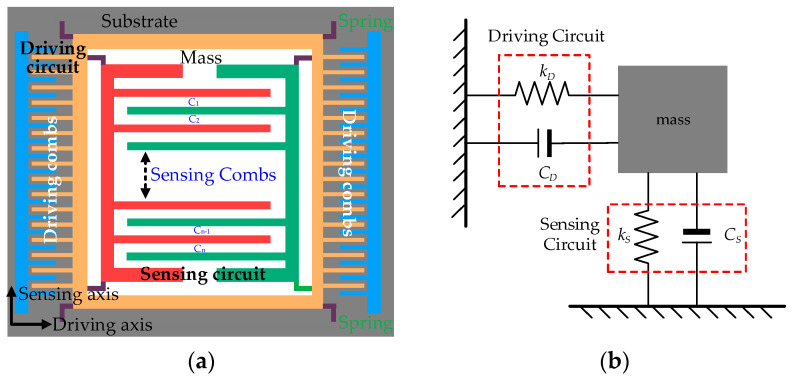
CMG’s composition and its basic principle. (**a**) Its composition; (**b**) its basic principle.

CMG is a type of miniaturized device, including a mass, a driving circuit, a sensing circuit and a substrate. With the technology of Micro Electro Mechanical Systems (MEMS), they are assembled as a module. Figure 1 shows its composition and basic principle.

Where *k_D_* is the stiffness of the driving suspension, *k_S_* is the stiffness of the sensing suspension, *C_D_* is the capacitance of the driving circuit, and *C_S_* is the capacitance of the sensing circuit. The sensing and driving circuits have movable interspersed combs, and adjacent sensing combs are separated by the opposite sensing comb. They can be abstracted as plate–capacitor pairs connected in sequence and in parallel, and work in a differential capacitance mode. The Coriolis force ***F_C_*** is an inertial force that acts on a moving mass in a rotating reference frame and ***F_C_*** = 2*m* (***v*** × ***ω***). *m* is the mass, ***v*** is its velocity relative to the rotating frame, and ***ω*** is the angular velocity of the frame. In CMG, the mass is driven to oscillate at a constant amplitude along the drive direction. When an external angular rate ***ω*** is applied, the resulting ***F_C_*** induces a vibration along the orthogonal sense direction, and the sensing combs displace away from their initial positions. The amplitude of this sense-mode vibration is proportional to the input angular rate ***ω***, which can be detected via capacitance variation. Assuming there are 2*n* combs in sensing combs, there are *n* air-gaps. From the plate capacitor’s formula, capacitance variation ∆*C* is expressed:

(1) $$\Delta C=\sum_{i=1}^{n}\left(C_{1}-C_{2}\right)=n\left(\frac{\varepsilon S_{0}}{d_{0}-\Delta d}-\frac{\varepsilon S_{0}}{d_{0}+\Delta d}\right)$$

where *ε* is the absolute dielectric constant of the dielectric, *S*_0_ is the overlap area between the sensing combs and *d*_0_ is its initial vertical distance, ∆*d* is the vertical distance variation at ***ω***, *C*_1_ is the total capacitance that narrow vertical distances form, and *C*_2_ is the one that wide vertical distances form. By measuring Δ*C*, the carriers’ angular rate ***ω*** can be demodulated using ***v***. Also, the stiffness of the sensing and driving circuits determines microstructural and micromotion consistency inside CMG. Ambient temperature, as an important factor to Si-based material’s stiffness, seems to be the root cause influencing microstructural and micromotion of CMG and brings the capacitance errors of the sensing combs as TDE ∆*E_CMG_*. The conventional TDE precise estimation model demonstrates microstructural deformation in a linear analytical method, and TDE is related to ambient temperature variation ∆*T*, its square ∆*T*^2^ and its square root ∆*T*^1/2^. So, it is expressed as [32]

(2) $$\Delta E_{CMG}\propto \left(\Delta T,\Delta T^{1/2},\Delta T^{2}\right)$$

#### 2.1.2. Modified TDE Precise Estimation Model Using Thermal-Induced Physical Characteristics Variation Analysis

1. Thermal-induced microstructural deformation

On the basis of the plate capacitor’s formula, the capacitance of the sensing combs is calculated with the microstructure sizes. However, due to the edge effect, the electric field lines of plate capacitors bend and diverge at the plates’ edge. It causes electric field lines to distribute unevenly, which makes the actual capacitance unequal to its calculated value. According to Kirchhoff’s classical edge effect theory, a correction formula of the plate capacitors *C*′ under the microstructure of CMG can be shown approximately as follows [33,34]:

(3) $$C^{\prime }=C_{0}\left[1+\frac{f\left(\varepsilon_{r}\right)d}{\pi c}\ln\left(\frac{4\pi c}{d}\right)\right]=\frac{\varepsilon_{0}^{\prime }\varepsilon_{r}bc}{d}\left[1+\frac{f\left(\varepsilon_{r}\right)d}{\pi c}\ln\left(\frac{4\pi c}{d}\right)\right]$$

where *C*_0_ is the capacitance without edge effect, *b* is the length of the plate, *c* is its width and *c* ≤ *b*, *d* is its distance, *ε*_0_′ is the vacuum dielectric constant, *ε_r_* is the relative dielectric constant, *f*(*ε_r_*) is a correction factor related to the relative dielectric constant and *f*(*ε_r_*) ≈ 1 for air medium.

(a) Under the condition of ambient temperature *T*_0_ and angular velocity *ω*_0_

**Figure 2:**
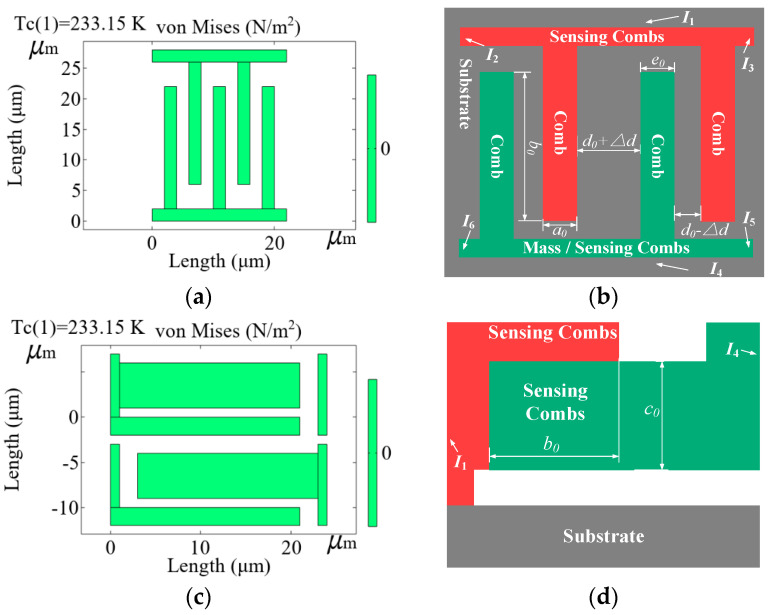
Microstructure of the sensing combs under *T*_0_ = −40 °C and *ω*_0_ in simulation. (**a**) Its top view in simulation; (**b**) its top view with sizes; (**c**) its side view in simulation; (**d**) its side view with sizes.

If the ambient temperature stays at *T*_0_, CMG’s microstructure is stable and the capacitance of the sensing combs stays constant. When the carriers rotate at *ω*_0_, the sensing combs displace in the y-axis. Due to the temperature dependence of Si-based material, varying ambient temperature causes 3D microstructural deformation, expanding as it rises or contracting as it falls, and those changes the air-gaps of the sensing combs. Based on that, a multi-physical field simulation software, COMSOL Multiphysics 6.2, simulates that situation. CMG is fabricated on a single-crystal silicon (110) wafer. The sensing combs are common values at a micrometer scale of 1~100 μm, the Si-based material’s thermal expansion coefficient is 2.4 × 10^−6^/°C, its Poisson’s ratio is 0.28, its elastic modulus is 175 GPa, and its density is 2330 kg/m^3^ [32]. CMG’s temperature range is taken as the control targets in simulation, usually from −40 °C to 85 °C, and −40 °C is set as the reference temperature. The mesh is generated using physics-controlled mesh sequence with normal element size setting, and the element size is 2 μm under convergence testing. A stationary study is applied to compute thermal expansion and stress distribution, and direct solver MUMPS is used with a relative tolerance of 1 × 10^−6^. Figure 2 shows the microstructure of the sensing combs under *T*_0_ = −40 °C and *ω*_0_ in simulation.

Where *a*_0_ is the thickness of the sensing comb at *T*_0_, *c*_0_ is its width at *T*_0_, *b*_0_ is the length of overlap area between the sensing combs at *T*_0_ and *S*_0_ = *b*_0_ *c*_0_, *d*_0_ is the distance between them at *T*_0_, *e*_0_ is the thickness of the sensing comb of the mass at *T*_0_, and *ε*_0_ is absolute dielectric constant of the dielectric at *T*_0_ and *ε*_0_ = *ε*_0_′*ε_r_*. The outer frame regions *I*_1_, *I*_2_, *I*_3_, *I*_4_, *I*_5_, and *I*_6_ are set as fixed constraints, and all other parts in Figure 2 are set as free boundaries. Distance variation at *ω*_0_ is ∆*d*, distance displacements *d*_1_ = *d*_0_ − ∆*d* and *d*_2_ = *d*_0_ + ∆*d*. *C*_1_′ is the total capacitance at *T*_0_ that narrow vertical distances form, and *C*_2′_ is the one at *T*_0_ that wide vertical distances form. Based on (1), ∆*C*′ is shown as follows:

(4) $$\begin{array}{l} \Delta C^{\prime }=\sum_{i=1}^{n}\left(C_{1}^{\prime }-C_{2}^{\prime }\right)=\frac{n\varepsilon_{0}^{\prime }\varepsilon_{r}b_{0}c_{0}}{d_{1}}\left[1+\frac{d_{1}}{\pi c_{0}}\ln\left(\frac{4\pi c_{0}}{d_{1}}\right)\right]-\frac{n\varepsilon_{0}^{\prime }\varepsilon_{r}b_{0}c_{0}}{d_{2}}\left[1+\frac{d_{2}}{\pi c_{0}}\ln\left(\frac{4\pi c_{0}}{d_{2}}\right)\right]\\ \;=n\varepsilon_{0}b_{0}c_{0}\left[\frac{2\Delta d}{d_{0}^{2}-\Delta d^{2}}+\frac{1}{\pi c_{0}}\ln\left(\frac{d_{0}+\Delta d}{d_{0}-\Delta d}\right)\right] \end{array}$$

(b) Under the condition of ambient temperature *T*_1_ and angular velocity *ω*_0_

**Figure 3:**
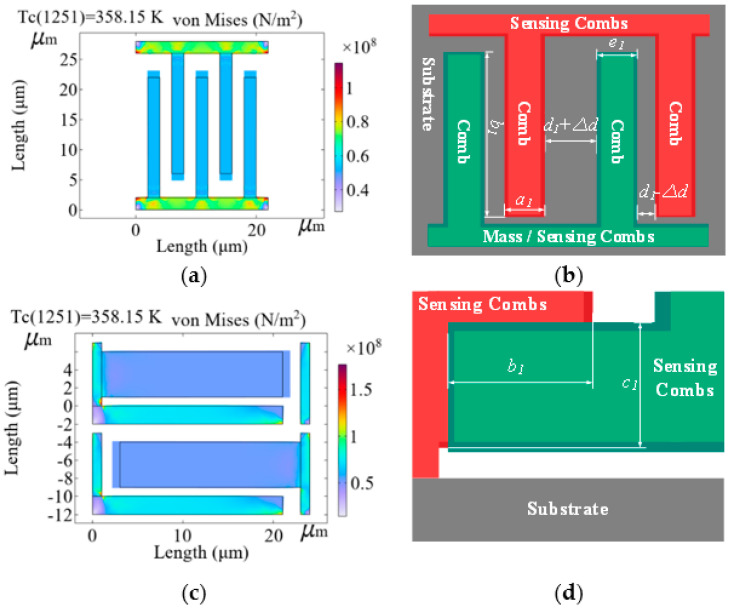
Microstructure of the sensing combs under *T*_1_ = 85 °C and *ω*_0_ in simulation. (**a**) Its top view in simulation; (**b**) its side view with sizes; (**c**) its top view in simulation; (**d**) its side view with sizes.

The sensing combs are of long-beam structure and include non-connecting and connecting ends. Non-connecting ends deform freely following the linear thermal expansion formula, and connecting ends’ thermal stress restricts free deformation, which causes a slightly local curved nonlinearity that is relatively weak and ignored [32]. Figure 3 shows the microstructure of the sensing combs under *T*_1_ = 85 °C and *ω*_0_ in simulation.

Where *a*_1_ is the thickness of the sensing comb at *T*_1_, *b*_1_ is the length of the overlap area between the sensing combs at *T*_1_, *c*_1_ is its width at *T*_1_ and *S*_1_ = *b*_1_*c*_1_, *d*_1_ is the distance between them, and *e*_1_ is the thickness of the sensing comb of the mass at *T*_1_. Due to the thermal expansion formula ∆*P* = *αP*_0_∆*T*, ∆*P* is the length variation at *T*_1_ and *P*_0_ is its length at *T*_0_, ambient temperature variation ∆*T* = *T*_1_ − *T*_0_, and *α* is the thermal expansion coefficient of Si-based material. From Figure 3, *a*_1_, *c*_1_ and *e*_1_ deform in two directions and *b*_1_ deforms in one direction, so they are shown as

(5) $$\begin{array}{cccccc} \Delta a=a_{0}\alpha \Delta T & \Delta b=b_{0}\alpha \Delta T & \Delta c=c_{0}\alpha \Delta T & \Delta e=e_{0}\alpha \Delta T & d_{1}^{\prime }=d_{1}-\frac{\Delta a}{2}-\frac{\Delta e}{2} & d_{2}^{\prime }=d_{2}-\frac{\Delta a}{2}-\frac{\Delta e}{2} \end{array}$$

In addition, owing to the fact that the absolute dielectric constant of the dielectric is temperature dependent, then that at *T*_1_ can be shown as *ε*_1_ [35]:

(6) $$\varepsilon_{1}=\varepsilon_{0}\left[1+\alpha_{\varepsilon }\left(T_{1}-T_{0}\right)\right]=\varepsilon_{0}\left(1+\alpha_{\varepsilon }\Delta T\right)$$

where α*_ε_* is the thermal expansion coefficient of the absolute dielectric constant of the dielectric at *T*_1_. Due to ∆*a* << *d*_0_ and ∆*e* << *d*_0_, ∆*C*″ can be shown as follows:

(7) $$\begin{array}{l} \Delta C^{\prime \prime }=\sum_{i=1}^{n}\left(C_{1}^{\prime \prime }-C_{2}^{\prime \prime }\right)=\frac{n\varepsilon_{1}b_{1}c_{1}}{d_{1}^{\prime }}\left[1+\frac{f\left(\varepsilon_{r}\right)d_{1}^{\prime \prime }}{\pi c_{1}}\ln\left(\frac{4\pi c_{1}}{d_{1}^{\prime }}\right)\right]-\frac{n\varepsilon_{1}b_{1}c_{1}}{d_{2}^{\prime }}\left[1+\frac{f\left(\varepsilon_{r}\right)d_{2}^{\prime }}{\pi c_{1}}\ln\left(\frac{4\pi c_{1}}{d_{2}^{\prime }}\right)\right]\approx n\varepsilon_{1}b_{1}c_{1}\left[\frac{d_{2}^{\prime }-d_{1}^{\prime }}{d_{1}^{\prime }d_{2}^{\prime }}+\frac{1}{\pi c_{1}}\ln\left(\frac{d_{2}^{\prime }}{d_{1}^{\prime }}\right)\right]\\ \;=n\varepsilon_{0}b_{0}c_{0}{\left(1+\alpha \Delta T\right)}^{2}\left(1+\alpha_{\varepsilon }\Delta T\right)\left[\frac{2\Delta d}{\left(d_{1}-\frac{\Delta a}{2}-\frac{\Delta e}{2}\right)\left(d_{2}-\frac{\Delta a}{2}-\frac{\Delta e}{2}\right)}+\frac{1}{\pi c_{1}}\ln\left(\frac{d_{0}+\Delta d-\frac{\Delta a}{2}-\frac{\Delta e}{2}}{d_{0}-\Delta d-\frac{\Delta a}{2}-\frac{\Delta e}{2}}\right)\right]\\ \;\approx n\varepsilon_{0}b_{0}c_{0}\left[\frac{2\Delta d}{d_{0}^{2}}{\left(1+\alpha \Delta T\right)}^{2}\left(1+\alpha_{\varepsilon }\Delta T\right)+\frac{\left(1+\alpha \Delta T\right)\left(1+\alpha_{\varepsilon }\Delta T\right)}{\pi c_{0}}\ln\left(\frac{d_{0}+\Delta d}{d_{0}-\Delta d}\right)\right] \end{array}$$

where *C*_1_″ is the total capacitance at *T*_1_ that forms narrow vertical distances, and *C*_2_″ is the one at *T*_1_ that forms wide vertical distances. Due to ∆*d* << *d*_0_, TDE ∆*E_CMG_* is deduced with (4) and (7):

(8) $$\begin{array}{l} \Delta E_{CMG}=\Delta C^{\prime \prime }-\Delta C^{\prime }\\ \;=n\varepsilon_{0}b_{0}c_{0}\left[\frac{2\Delta d}{d_{0}^{2}}{\left(1+\alpha \Delta T\right)}^{2}\left(1+\alpha_{\varepsilon }\Delta T\right)+\frac{\left(1+\alpha \Delta T\right)\left(1+\alpha_{\varepsilon }\Delta T\right)}{\pi c_{0}}\ln\left(\frac{d_{0}+\Delta d}{d_{0}-\Delta d}\right)\right]-n\varepsilon_{0}b_{0}c_{0}\left[\frac{2\Delta d}{d_{0}^{2}-\Delta d^{2}}+\frac{1}{\pi c_{0}}\ln\left(\frac{d_{0}+\Delta d}{d_{0}-\Delta d}\right)\right]\\ \;=n\varepsilon_{0}b_{0}c_{0}\left[\frac{2\Delta d}{d_{0}^{2}}{\left(1+\alpha \Delta T\right)}^{2}\left(1+\alpha_{\varepsilon }\Delta T\right)-\frac{2\Delta d}{d_{0}^{2}}+\frac{\left(\alpha +\alpha_{\varepsilon }\right)\Delta T+\alpha \alpha_{\varepsilon }\Delta T^{2}}{\pi c_{0}}\ln\left(\frac{d_{0}+\Delta d}{d_{0}-\Delta d}\right)\right]\\ \;\propto \left(\Delta T,\Delta T^{2},\Delta T^{3}\right) \end{array}$$

From (8), ∆*E_CMG_* is related to ∆*T* and its square ∆*T*^2^ plus its cube ∆*T*^3^. Then, the conventional model can be modified as follows:

(9) $$\Delta E_{CMG}\propto \left(\Delta T,\Delta T^{1/2}\Delta T^{2},\Delta T^{3}\right)$$

2. Thermal-induced Si-based material’s stiffness deterioration

From Figure 1, the mass is driven by the driving circuit at resonant frequency *f_d_*:

(10) $$f_{d}=\frac{1}{2\pi }\sqrt{\frac{k_{d}}{m}}$$

where *k_d_* is the elastic coefficient in the driving direction, which is stiffness, and *m* is the mass. Ambient temperature variation also causes thermal expansion or contraction of the spring of the driving circuit, which changes the elasticity coefficient and the stiffness. So, *k_d_* is related to the elastic modulus *E* and the geometric dimensions of the springs, and is shown as

(11) $$k_{d}\propto \frac{Ewt^{3}}{l^{3}}$$

where *w* is the width of the spring, *t* is its thickness, and *l* is its length. By substituting (11) into (12), we can obtain as follows:

(12) $$f_{d}\propto \frac{1}{2\pi }\sqrt{\frac{Ewt^{3}}{l^{3}}\frac{1}{m}}=\frac{1}{2\pi \sqrt{m}}\sqrt{\frac{Ewt^{3}}{l^{3}}}$$

**Figure 4:**
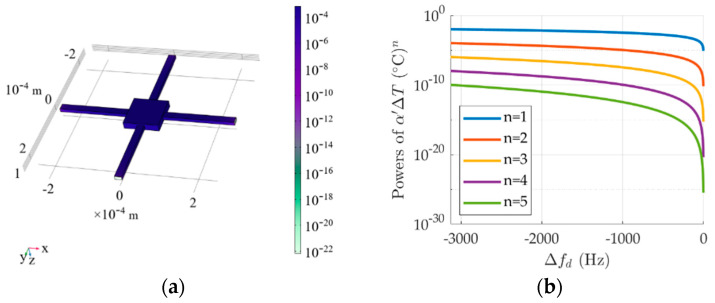
Microstructural deformation at 85 °C in simulation and the relationship between ∆*T^n^* and ∆*f_d_*. (**a**) Microstructural deformation; (**b**) the relationship between ∆*T^n^* and ∆*f_d_*.

Because Si-based material is temperature dependent, *E* is related to ∆*T* as follows [16]:

(13) $$E_{1}=E_{0}\left(1+\alpha_{E}\Delta T\right)$$

where *α_E_* is the temperature coefficient of the elastic modulus *E*. Based on (12) and (13), the resonant frequency error ∆*f_d_* = *f_d_*(*T*_1_) − *f_d_*(*T*_0_) is shown as follows:

(14) $$\Delta f_{d}\propto \frac{1}{2\pi \sqrt{m}}\sqrt{\frac{E_{1}w_{1}t_{1}^{3}}{l_{1}^{3}}}-\frac{1}{2\pi \sqrt{m}}\sqrt{\frac{E_{0}w_{0}t_{0}^{3}}{l_{0}^{3}}}=\frac{1}{2\pi \sqrt{m}}\sqrt{\frac{E_{0}\omega_{0}t_{0}^{3}}{L_{0}^{3}}}\left[\sqrt{1+\left(\alpha_{E}+\alpha \right)\Delta T}-1\right]$$

where *w*_0_ is the width of the spring of the driving circuit at *T*_0_ and *w*_1_ is that at *T*_1_, *t*_0_ is its thickness at *T*_0_ and *t*_1_ is that at *T*_1_, *l*_0_ is its length at *T*_0_ and *t*_1_ is that at *T*_1_, *E*_0_ is its elastic modulus at *T*_0_ and *E*_1_ is that at *T*_1_. Using the Taylor expansion, (14) is deduced as follows:

(15) $$\Delta f_{d}\propto \left[\frac{\alpha^{\prime }\Delta T}{2}-\frac{{\left(\alpha^{\prime }\Delta T\right)}^{2}}{8}+\frac{{\left(\alpha^{\prime }\Delta T\right)}^{3}}{16}-\frac{5{\left(\alpha^{\prime }\Delta T\right)}^{4}}{128}+\ldots \right]\propto f\left[{\left(\alpha^{\prime }\Delta T\right)}^{n}\right]$$

where *n* is Taylor expansion’s order (*n* = 1,2,3…), *α*′ = *α_E_* + *α*. To show the effect of ∆*T_n_* on ∆*f_d_*, a theoretical model of CMG in Figure 1 is simulated with COMSOL. The masses’ sizes are 100 μm × 100 μm × 20 μm (Length × Width × Height), long beams’ sizes are 200 μm × 20 μm × 10 μm. Other parameters are referred to in Figure 2. When ambient temperature rises from −40 °C to 85 °C at the interval of 0.1 °C, Figure 4 shows microstructural deformation of GMG at 85 °C in simulation and the relationship between ∆*T^n^* and ∆*f_d_*.

As shown in Figure 4, if the ambient temperature rises, CMG’s mass and long beams deform, and its resonant frequency decreases. Owing to the fact that *α_E_* is −50 ppm/°C~−80 ppm/°C and *α* is 2.4 × 10^−6^/°C, there is a weaker correlation between (*α*′∆*T*)*^n^* and ∆*f_d_* as *n* increases. It means that a higher power of *α*′∆*T* has an insignificant effect on reducing the resonant frequency difference. If the Taylor expansion performs on (15) more than fourth, the fourth power of *α* is little enough compared to the fourth power of ∆*T*, and higher powers are useless for ∆*f_d_*. It is needless to perform Taylor expansion more than the fourth, which illustrates that ∆*T^4^* plays an important role in ∆*f_d_*. Based on (9), the modified model can be further improved as follows:

(16) $$\Delta E_{CMG}\propto \left(\Delta T,\Delta T^{1/2},\Delta T^{2},\Delta T^{3},\Delta T^{4}\right)$$

### 2.2. Parameter Identification of Modified TDE Precise Estimation Model Using BO-LSTM

Ambient temperature is a typical physical quantity of time series characteristics, and its current value is related to those in the past and future. Parameter identification methods proficient in predicting time series are needed to identify the modified model properly. LSTM is a kind of time Recurrent Neural Networks (RNNs), including an input gate, forget gate, output gate, and memory unit. It can control information flow by gating mechanisms to eliminate gradient vanishing and its exploding in a long sequence training [36]. According to the universal approximation theorem, a sufficiently complex LSTM can approximate any continuous nonlinear function precisely [36]. Although it fits complex nonlinearity in time series by recursion and gating mechanisms of the hidden state, some points need to be focused on:

(1) Sufficient training data are needed. The more they are, the more precisely the target nonlinearity is described.
(2) By means of regularization and cross-validation, LSTM suppresses the noise in the target signals to avoid overfitting and increases its fitting accuracy in real-time as well.
(3) Better hyperparameter tuning fits complex nonlinearity more precisely, and it is essential for the fitting performance.

So, (1) and (2) can be overcome by its theory and extensive experiments, and hyperparameter tuning is the key to identifying LSTM accurately and properly. Usually, it relies on extensive training for the optimal hyperparameters, such as much time and enough resources, plus many tries, and then optimizing hyperparameter tuning is essential. BO suits the scenarios with the computationally expensive black box functions and the derivatives obtained difficultly [37,38]. It has a probabilistic surrogate model using a Gaussian Process (GP) and a collection function determining the next evaluation point. It builds a target function by GP, such as LSTM’s performance of layers’ number, hidden layers’ size, learning rate, dropout rate and so on, then minimizing the acquisition function determines the next evaluated hyperparameters. Based on that, BO-LSTM can be established as follows:

**Step 1**. Define the hyperparameter space of LSTM *θ* as shown:

(17) $$\theta =\left(\eta ,h,q,p,s,b,\lambda \right)$$

where *η* is the learning rate of LSTM, *h* is its hidden layer size, *q* is its number of layers, *p* is its dropout rate, *s* is its sequence length, *b* is its batch size, and *λ* is L2 regularization.

**Step 2**. Model target function *f*(*θ*) with GP, then we obtain:

(18) $$f\left(\theta \right)\sim GP\left[m\left(\theta \right),k\left(\theta ,\theta^{\prime }\right)\right]$$

where *m*(*θ*) is mean function, *k*(*θ*, *θ′*) is covariance function. If *t′* estimated data is obtained, the data set *D*_1:_*_t_*_′_ includes the first observation to the *t*′ observation as follows:

(19) $$D_{1:t^{\prime }}={\left\{\left(\theta_{i},L_{i}\right)\right\}}_{i=1}^{t^{\prime }}$$

where *L_i_* = *m*(*θ*) + *ε_i_* and *ε_i_*~*N*(0, *σ_n_*^2^).

**Step 3**. If the joint distribution of finite points θ_*_ is GP, then:

(20) $$\left[\begin{array}{c} f\left(\theta \right)\\ f\left(\theta_{*}\right) \end{array}\right]∼N\left(0,\left[\begin{array}{cc} K+\sigma_{n}^{2}I & k_{*}\\ k_{*} & k\left(\theta_{*},\theta_{*}\right) \end{array}\right]\right)$$

where *k_ij_* = *k*(*θ_i_*, *θ_j_*)∈*K*, k_*_ = [*k*(*θ*_1_, *θ*), …, *k*(*θ_t′_*, *θ*)]*^T^*, *I* is a unit array. Predicted distribution of new hyperparameter θ_*_ is GP *p* as follows:

(21) $$p\left[f\left(\theta_{*}\right)|D_{1:t^{\prime }}\right]\sim N\left[\mu_{t^{\prime }}\left(\theta_{*}\right),\sigma_{t^{\prime }}^{2}\left(\theta_{*}\right)\right]$$

where *μ_t′_*(θ_*_) = k_*_^T^(*K* + *σ_n_*^2^*I*)^−1^*L_i_*, *σ_t′_*^2^(θ_*_) = *k*(θ_*_,θ_*_)-k_*_^T^(*K* + *σ_n_*^2^*I*)^−1^k_*_.

**Step 4**. *α_EI_*(*θ*) is set as the acquisition function and shown as follows:

(22) $$\alpha_{EI}\left(\theta \right)=E\left\{\max\left[0,f^{+}-f\left(\theta \right)\right]\right\}$$

where *f*^+^ =*min_i_*_=1,…,_*_t′_f*(*θ_t′_*), which is the current optimal observation loss. We can obtain its analytical solution *α_EI_*(*θ*) as follows:

(23) $$\alpha_{EI}\left(\theta \right)=\left\{\begin{array}{cc} \Delta \Phi \left(z\right)+\sigma_{t^{\prime }}\left(\theta \right)\phi \left(z\right) & \sigma_{t^{\prime }}\left(\theta \right)>0\\ 0 & \sigma_{t^{\prime }}\left(\theta \right)=0 \end{array}\right.$$

where *z* = ∆/*σ_t′_*(*θ*) = [*f*^+^ − *μ_t′_*(*θ*)]/*σ_t′_*(*θ*) and predict improvement ∆ = *f*^+^ − *μ_t′_*(*θ*), *σ_t′_*(*θ*) is the posterior standard deviation of GP at *θ*, *ϕ* follows a standard normal probability density function and Φ follows its cumulative distribution function. Improvement probability *α_IP_*(*θ*) is shown:

(24) $$\alpha_{IP}\left(\theta \right)=P\left[f\left(\theta \right)<f^{+}\right]=\Phi \left[\frac{f^{+}-\mu_{t^{\prime }}\left(\theta \right)}{\sigma_{t^{\prime }}\left(\theta \right)}\right]$$

where *P* is the probability function. Its confidence upper bound *α_CUB_* is shown as follows:

(25) $$\alpha_{CUB}\left(\theta \right)=-\mu_{t^{\prime }}\left(\theta \right)+K_{t^{\prime }}\sigma_{t^{\prime }}\left(\theta \right)$$

where *K_t′_* = 2log[*t′*^(^*^d^*^′/2+2)^*π*^2^/3*σ_t′_*]^1/2^, *d*′ is dimension of *θ*.

**Step 5**. LSTM is trained as a black box function and its next hyperparameters *θ^k+1^* are

(26) $$\theta^{k+1}=\theta^{k}-\zeta \nabla_{\theta }f\left(\theta^{k}\right)$$

where $\nabla_{\theta }$ is *θ*′s gradient, *θ ^k+^*^1^ are LSTM’s current hyperparameters, and *ζ* is the learning rate. Its verify loss *f*(*θ*) can be calculated as follows:

(27) $$f\left(\theta \right)=\frac{1}{N}\sum_{i=1}^{N}\psi \left[y_{i}^{\prime },f_{\theta }\left(x_{i}\right)\right]$$

where *N* is the data volume of the verification set, *y_i_′* is data in it, *f_θ_*(*x_i_*) is the values of the target function *f_θ_* at input point *x_i_* in the verification set, and *ψ* is an acquisition function like **EI**(*x*).

**Step 6**. Based on all the mentioned, the input vector is set as *x_t_* = [Δ*T*, Δ*T*^1/2^, Δ*T*^2^, Δ*T*^3^, Δ*T*^4^] and the output vector is set as *h_t_* = Δ*E′_CMG_*. The training data *D*_0_, including *x_t_* and *h_t_,* is split as the training set *D*_1_, which is 80% of *D*_0_, and the validation set *D*_2_, which is 20% of *D*_0_. The training set trains BO-LSTM, and the validation set verifies if the training results meet accuracy requirements with verify loss Root Mean Square Error (RMSE).

**Step 7**. The hyperparameters from (17) are chosen, and BO-LSTM is trained under them with MATLAB (R2023a). The iterative process for training is done continuously, and in each iteration *GP* is updated with existing observation data, including the training and validation sets, hyperparameters and their corresponding loss RMSE. The next hyperparameters are chosen by optimizing the acquisition function, then they will be evaluated and added to the existing observation data. When the minimum RMSE appears, the iterative process stops and the optimal hyperparameters are obtained. Based on that, LSTM’s hyperparameters are optimized, and the modified model can be identified precisely and coded as an in-house script using standard MATLAB functions, which is shown as follows:

(28) $$\Delta E_{CMG}=BO-LSTM\left(\Delta T,\Delta T^{1/2},\Delta T^{2},\Delta T^{3},\Delta T^{4}\right)$$

## 3. Experiment and Analysis

### 3.1. Accurate TDE Test Methodology

Based on (28), the more accurately the TDE of CMG is tested, the more precisely the modified model is identified. So, testing TDE accurately is a key prerequisite; a proper TDE test method is needed, and some points need to be focused on:

(a) Good heat conduction treatment

Good heat conduction treatment transfers the heat to CMG completely, which reduces the temperature gradient to ensure the experimental results’ reliability, such as thermal conductive rubber, thermal grease, liquid metals and so on.

(b) Precise temperature measurement

Measuring the ambient temperature of CMG precisely ensures describing its environmental adaptability wholly. A temperature sensor of a precise temperature measurement system is installed on CMG closely to reduce the temperature gradient. To test intact experimental results, its accuracy is more accurate than ∆*T* by over 2 times and its frequency is higher than CMG’s output.

(c) Proper temperature control interval

From CMG’s datasheets, TDE can be calculated roughly as ∆*E′* = *γ*∆*T* + *β*∆*T*. ∆*E′* is a little smaller than TDE because some non-statistical errors are ignored, *γ* is “zero-rate level change vs. temperature” and *β* is “sensitivity change vs. temperature”. If ambient temperature varies suddenly, it is likely that TDE is greater than CMG’s sensitivity ∆*E_S_*. To test TDE accurately, set ∆*E_S_*≈∆*E′*, and the temperature controlling interval ∆*T_I_* is shown [32]:

(29) $$\Delta T_{I}\leq \frac{\Delta E_{S}}{\gamma +\beta }$$

(d) Reasonable temperature control period

The thermal chamber is used to test TDE, and it is designed as a cuboid with closed insulation and a front door. It integrates a temperature controlling system and sets controlling units on each surface. Also, it installs a rate table to offer the target angular velocity.

Usually, a thermal chamber is designed as a cube whose size is *L* × *L* × *L*, and the control target of each surface is set as *T_b_*. To avoid an incomplete heat conduction process to bring imprecise TDE, a temperature controlling period *t_s_* is formed [32]:

(30) $$t_{s}=\frac{1}{k_{h}T_{b}}C\rho L^{2}\left(T_{b}-T_{0}\right)$$

where *C* is the specific heat capacity of air in a thermal chamber in a closed condition, and *ρ* is its air density, *k_h_* is the coefficient of heat conductivity, *T*_0_ is the initial ambient temperature and *T_b_* is the final one. To heat the thermal chamber uniformly, set the temperature controlling period as *t_p_* ≥ *t_s_*. CMG, as a test object, is a commercial device, L3GD20H, manufactured by STMicroelectronics. From the datasheet, the key characteristics include a user-selectable measurement range of ±245/±500/±2000 dps, a sensitivity of 8.75–70.00 mdps/digit, and a digital zero-rate level of ±25 dps. In addition, it is housed in a plastic LGA package with the dimensions of 4.0 mm × 4.0 mm × 1.1 mm (length × width × height). Its parameters ∆*E_S_* = 8.75 mdps/digit, *γ* = ±0.04 dps/°C, and its operating temperature range is −40~85 °C [32]. So, *β* is shown:

(31) $$\beta =2\%/{}^{\circ}C\times 8.75\;mdps\times \frac{85\;{}^{\circ}C-\left(-40\;{}^{\circ}C\right)}{2}=10.9375\;mdps/{}^{\circ}C$$

According to (29), ∆*T_I_* can be calculated and shown as follows:

(32) $$\Delta T_{I}\leq \frac{8.75\;mdps/\mathrm{digit}}{0.04\;dps/{}^{\circ}C\;\times \frac{245\;dps}{2000\;dps}+10.9375\;mdps/{}^{\circ}C\;}\approx 0.55\;{}^{\circ}C$$

To simplify the testing steps, set ∆*T_I_* = 0.5 °C. Then, thermal chamber SET-Z-021 is utilized to test L3GD20H, and its parameters are *C* = 1.005 kJ/(kg × K), *k_h_* = 0.026 W/m°C, *L* = 0.6 m, and *ρ* = 1.293 kg/m^3^. Its operating temperature range is −40 °C~85 °C, and (30) is calculated as follows:

(33) $$t_{s}\approx 25.766\;s$$

To simplify test steps and reserve a margin for stable heat conduction, set *t_s_* = 30 s. L3GD20H is tested in the temperature experiments, and its temperature is measured by a temperature measurement system whose measuring accuracy is ±0.03 °C and measuring frequency is 10 Hz. The temperature experiment is designed as follows:

**Step 1**: L3GD20H is installed on the rate table. Its measuring direction is parallel to the rate table, and its referenced true value is the angular velocity of the rate table. Under a condition of static base, its referenced true value *ω*_0_ = 0 dps.

**Step 2**: Attach temperature sensors on top of L3GD20H closely. The wireless transmission module sends the experimental results, including temperature *T_CMG_* and L3GD20H’s output *D_CMG_* at 10 Hz. Meanwhile, PC receives all of the experimental results in a timely manner.

**Step 3**: Cool L3GD20H down to −40 °C, and record *T_CMG_* and *D_CMG_* for 0.5 h until the ambient temperature measured by the temperature sensors stays stable.

**Step 4**: Heat L3GD20H up to 85 °C at 60 °C/h, i.e., 0.5 °C per 30 s. Stop the experiment until *T_CMG_* stabilizes at 85 °C for 0.5 h; meanwhile, PC receives all of the experimental results.

**Step 5**: Repeat step (2) to step (4) five times and record the experimental results. In addition, normalize them to avoid underfitting or overfitting due to the large or small values for easy training.

**Figure 5:**
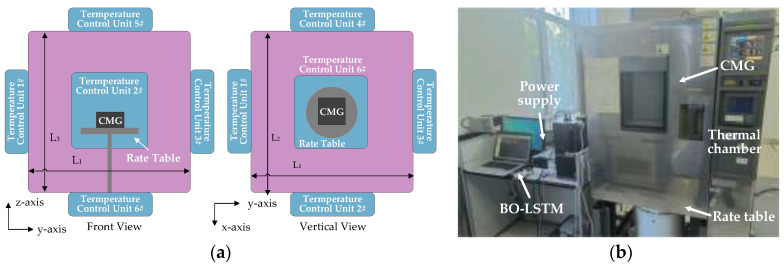
Schematic diagram of TDE test. (**a**) Its front and vertical views; (**b**) test site.

**Figure 6:**
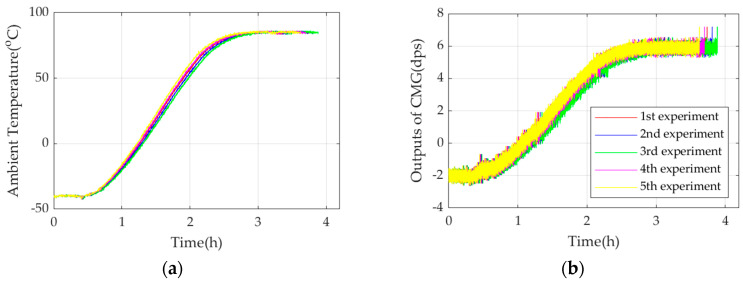
The experimental results of L3GD20H in five experiments. (**a**) Ambient temperature; (**b**) L3GD20H’s outputs.

Figure 5 shows a schematic diagram of the TDE test. Figure 6 shows the experimental results of L3GD20H in five experiments.

### 3.2. Effect Comparison of Conventional and Complete TCQs

From Figure 6, when the ambient temperature rises from −40 °C to 85 °C, CMG outputs in a similar trend. Ambient temperature is stable for 0.5 h at the beginning, which is the referenced temperature, and the referenced output of CMG is 0 dps. To demonstrate that complete TCQ is much more effective than conventional TCQ on TDE precise estimation, they are established based on (2) as follows:

(34) $$\begin{array}{cc} \Delta E_{CMG}=RBFNN\left(\Delta T,\Delta T^{1/2},\Delta T^{2}\right) & \Delta E_{CMG}^{\prime }=RBFNN\left(\Delta T,\Delta T^{1/2},\Delta T^{2},\Delta T^{3},\Delta T^{4}\right) \end{array}$$

**Figure 7:**
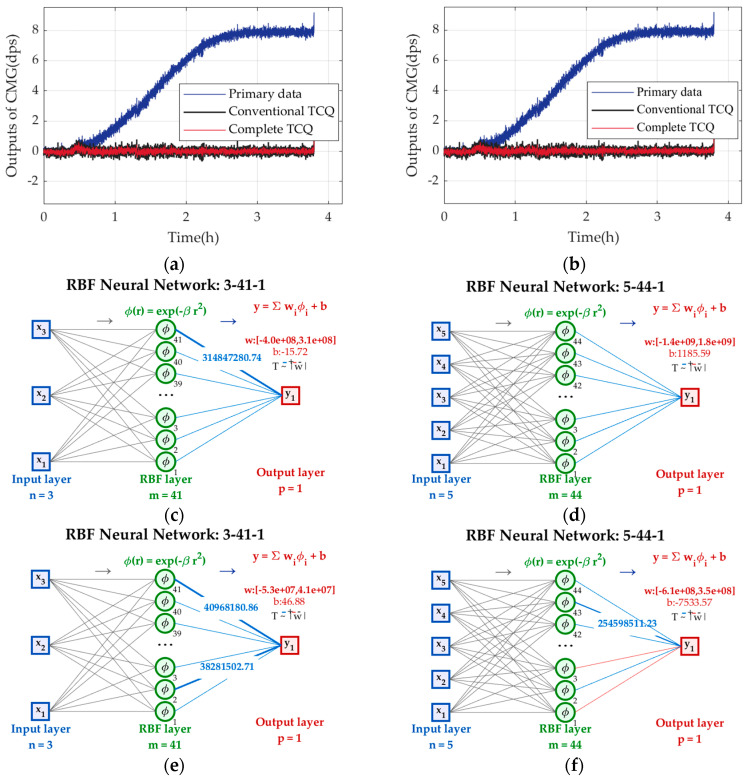
The primary data and those compensated by conventional and complete TCQs in two sets. (**a**) Experimental results in set 1; (**b**) experimental results in set 2; (**c**) RBFNN based on conventional TCQ in set 1; (**d**) RBFNN based on complete TCQ in set 1; (**e**) RBFNN based on conventional TCQ in set 2; (**f**) RBFNN based on complete TCQ in set 2.

Two sets of experimental results in Figure 6 are chosen to train (34), and Figure 7 shows the primary data and those compensated by (34) in two sets, plus their applications in CMG.

To show the estimation performance, bias stability (BS) is a key index to describe the extent to which CMG’s output varies or drifts around its mean values in constant conditions. It is calculated by the Allan variance, which separates the contributions of different types of noise to the overall error, like white noise, flicker noise and random walk, which is shown as

(35) $$\sigma_{av}^{2}\left(\tau \right)=\frac{1}{2\left(M-1\right)}\sum_{k=1}^{M-1}{\left[\Omega_{k+1}\left(\tau \right)-\Omega_{k}\left(\tau \right)\right]}^{2}$$

where *τ* is clustering time, *M* is the total number of clusters in *τ*, *σ_av_*(*τ*) is the Allan variance standard deviation, and Ω*_k_* is the average of the *k*th cluster. A smaller *σ_av_*(*τ*) shows a more precise TDE estimate. BS of the primary data is *BS*_1_, BS compensated by conventional TCQ is *BS*_2_, BS done by complete TCQ is *BS*_3_, *Q*_1_ = *BS*_2_/*BS*_1_, *Q*_2_ = *BS*_3_/*BS*_1_. They are shown in Table 1.

In Figure 7, after being compensated, CMG’s outputs are stable with small fluctuation around *ω*_0_ = 0 dps, but those compensated by complete TCQ are more stable. From Table 1, complete TCQ reduces bias stability by 7%, evenly better. So, it shows that complete TCQ plays a more crucial role in describing the root cause of TDE of CMG and offers a better reference for TDE precise estimation.

### 3.3. Effect Comparison of RBFNN and BO-LTSM

As a key way for LSTM’s optimal hyperparameters, manual tuning relies heavily on experience and intuition. According to (17), the hyperparameter space is usually shown in Table 2.

**Table 2:** Hyperparameter space of LSTM.

Hyperparameters	*η*	*h*	*l*	*p*	*s*	*b*	*λ*
Space	[0.001, 0.1]	[30, 150]	[1, 2]	[0.1, 0.5]	[15, 50]	[16, 64]	[0.007, 0.003]

If the minimum unit of the hyperparameters is set as their minimum divided value, hyperparameter combinations *N_hc_* are shown as follows:

(36) $$N_{hc}=\Delta \eta \Delta h\Delta l\Delta p\Delta s\Delta b\Delta \lambda =100\times 120\times 2\times 5\times 35\times 49\times 5=1.029\times 10^{9}$$

where ∆*η*, ∆*h*, ∆*l*, ∆*p*, ∆*s*, ∆*b*, and ∆*λ* are minimum divided units of *η*, *h*, *l*, *p*, *s*, *b* and *λ*. It needs 1.029 × 10^9^ tries to search all hyperparameter combinations, and that is a large workload and impossible to fulfil in a good convergence, repeatability and search efficiency. BO-LSTM avoids needless hyperparameter combinations from RMSE’s convergence, and search efficiency and repeatability are enhanced to save time and resources. To show that BO-LSTM identifies the TDE estimation model better than RBFNN, they are used in (2) and compared:

(37) $$\begin{array}{cc} \Delta E_{CMG}=RBFNN\left(\Delta T,\Delta T^{1/2},\Delta T^{2}\right) & \Delta E_{CMG}^{\prime }=BO-LSTM\left(\Delta T,\Delta T^{1/2},\Delta T^{2}\right) \end{array}$$

**Figure 8:**
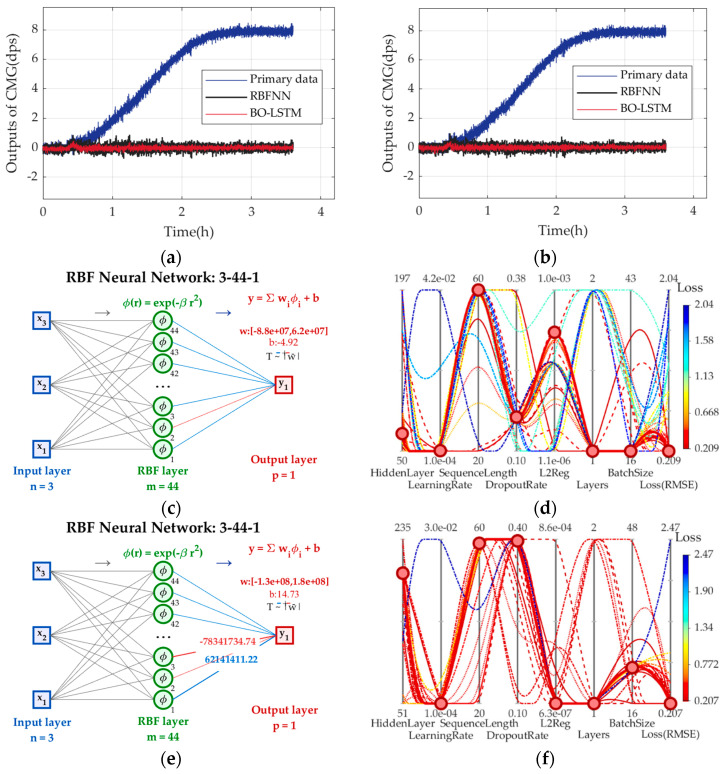
The primary data compensated by RBFNN and BO-LSTM in two sets. (**a**) Experimental results in set 1; (**b**) experimental results in set 2; (**c**) RBFNN in set 1; (**d**) hyperparameter tuning of BO-LSTM in set 1; (**e**) RBFNN in set 2; (**f**) hyperparameter tuning of BO-LSTM in set 2.

Two sets of experimental results in Figure 6 are chosen to train (37), and Figure 8 shows the primary data and those compensated by (37), plus their applications in CMG. Table 3 shows BO-LSTM’s optimal hyperparameters in two sets. BS of the primary data is *BS*_4_, BS compensated by RBFNN is *BS*_5_, BS compensated by BO-LSTM is *BS*_6_, *Q*_3_ = *BS*_5_/*BS*_4_, *Q*_4_ = *BS*_6_/*BS*_4_. They are shown in Table 4.

From Figure 8, CMG after compensated stabilize at *ω*_0_ = 0 dps, and BO-LSTM keeps GMG’s outputs more stable. After more than 40k iterations, EBFNN and BO-LSTM based on the conventional TCQ are built, and BO-LSTM needs fewer tries than manual tuning to increase training efficiency. From Table 4, it can reduce the stability of CMG by 7.69% evenly, so it can identify TDE precise estimation model more precisely and more effectively.

### 3.4. Effect Comparisons of Conventional and Modified Models

**Figure 9:**
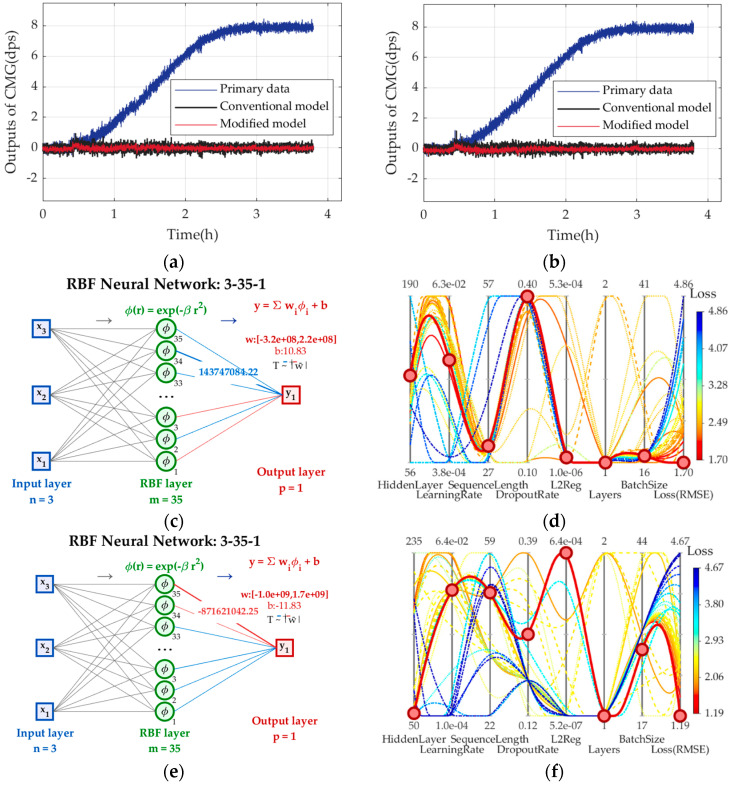
The primary data compensated for by conventional and modified models in two sets. (**a**) Experimental results in set 1; (**b**) experimental results in set 2; (**c**) RBFNN in set 1; (**d**) hyperparameter tuning of BO-LSTM in set 1; (**e**) RBFNN in set 2; (**f**) hyperparameter tuning of BO-LSTM in set 2.

To show the modified model estimates a more precise TDE, (2) and (28) are built with two sets of experimental results. Figure 9 shows the primary data and those compensated by them, plus their applications. BO-LSTM’s optimal hyperparameters are shown in Table 5. BS of the primary data is *BS*_7_, BS compensated by the conventional model is *BS*_8_, BS compensated by the modified model is *BS*_9_, *Q*_5_ = *BS*_8_/*BS*_7_, *Q*_6_ = *BS*_9_/*BS*_7_. They are shown in Table 6.

(38) $$\begin{array}{cc} \Delta E_{CMG}=RBFNN\left(\Delta T,\Delta T^{1/2},\Delta T^{2}\right) & \Delta E_{CMG}^{\prime }=BO-LSTM\left(\Delta T,\Delta T^{1/2},\Delta T^{2},\Delta T^{3},\Delta T^{4}\right) \end{array}$$

From Figure 9, CMG after compensation by conventional and modified models runs stably. From Table 6, they reduce the bias stability of CMG, and the modified model reduces bias stability significantly, by 16% evenly, compared to the conventional model. So, the modified model can estimate the TDE of CMG more precisely to improve bias stability markedly.

### 3.5. Verification of Modified TDE Model Based on BO-LSTM

**Figure 10:**
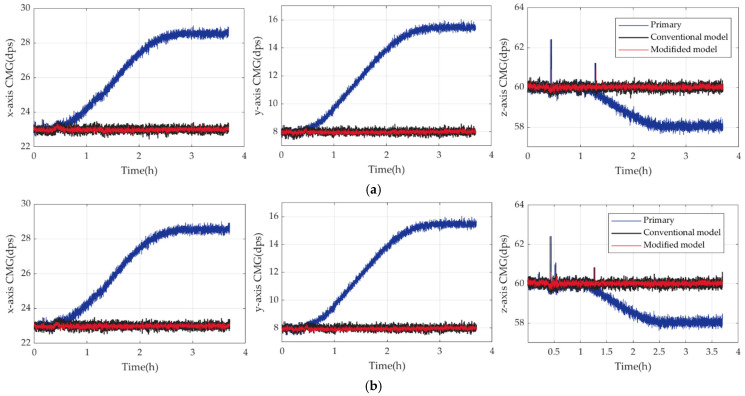
The primary data and those compensated by the conventional and modified models in two verifications in three axes. (**a**) Experimental results in verification 1; (**b**) experimental results in verification 2.

To verify TDE estimation accuracy and the universality of the modified model, another typical CMG I3G4250D is tested. Its conventional model using RBFNN and (∆*T*, ∆*T*^1/2^, ∆*T*^2^) plus its modified one based on BO-LSTM and (∆*T*, ∆*T*^1/2^, ∆*T*^2^, ∆*T*^3^, ∆*T*^4^) are established. To ensure the experimental results’ credibility, the referenced angular velocities are random, *ω_ref_^x^* = 23 dps in the *x*-axis, *ω_ref_^y^* = 8 dps in the *y*-axis, and *ω_ref_^z^* = 60 dps in the *z*-axis. Figure 10 shows the experimental results in two verifications. BS of the primary data is *BS*_10_, BS compensated by the conventional model is *BS*_11_, and BS compensated by the modified model is *BS*_12_, *Q*_7_ = *BS*_11_/*BS*_10_, *Q*_8_ = *BS*_12_/*BS*_10_. They are shown in Table 7.

As shown in Figure 10, after being compensated by the conventional and modified models, CMG runs more stably than the primary data. From Table 7, the conventional model reduces their bias stability to 1.53% of the primary data evenly; by contrast, the modified one reduces that to 1.19%. The modified model enhances CMG better by 20.73% than the conventional one in bias stability. So, the modified model can estimate TDE more precisely with much more perfect universality to improve the bias stability of CMG significantly.

## 4. Conclusions

In the paper, the complete TCQ (∆*T*, ∆*T*^1/2^, ∆*T*^2^, ∆*T*^3^, ∆*T*^4^) is traced precisely by analyzing microstructural deformation in CMG- and Si-based materials’ stiffness deterioration, and a modified TDE precise estimation model is built. Then, LSTM is applied to its parameter identification owing to the time series characteristics of TDE and TCQ, and BO is introduced to give a good guide for LSTM’s optimal hyperparameters. By comparison with the conventional model based on RBFNN plus (∆*T*, ∆*T*^1/2^, ∆*T*^2^), the modified model based on BO-LSTM plus (∆*T*, ∆*T*^1/2^, ∆*T*^2^, ∆*T*^3^, ∆*T*^4^) can reduce bias stability of CMG by 20% evenly, which means TDE of CMG can be estimated precisely and timely to decouple temperature dependence of Si-based material and enhance environmental adaptability of CMG significantly in complex conditions. Still, it is universal and robust, and, thus, can be applied widely.

## Figures and Tables

**Table 1:** BSs and improvement of conventional and complete TCQs.

Set No.	*BS*_1_ (dps)	*BS*_2_ (10^−2^ dps)	*BS*_3_ (10^−2^ dps)	*Q*_1_ (10^−2^)	*Q*_2_ (10^−2^)	|*Q*_2_ − *Q*_1_|/*Q*_1_
1	3.2104	4.1653	3.8236	1.2974	1.1910	8.20%
2	3.2101	4.0180	3.8054	1.2517	1.1855	5.29%

**Table 3:** BO-LSTM’S optimal hyperparameters.

Set No.	*η*	*h*	*q*	*p*	*s*	*b*	*λ*	Iterations
1	1.0 × 10^−4^	73	1	0.19	60	16	2.3 × 10^−6^	45,000
2	1.0 × 10^−4^	198	1	0.40	60	24	6.3 × 10^−7^	40,000

**Table 4:** BSs and improvement of RBFNN and BO-LSTM.

Set No.	*BS*_4_ (dps)	*BS*_5_ (10^−2^ dps)	*BS*_5_ (10^−2^ dps)	*Q*_3_ (10^−2^)	*Q*_4_ (10^−2^)	|*Q*_4_ − *Q*_3_|/*Q*_3_
1	3.1845	3.6000	3.3325	1.1294	1.0465	7.34%
2	3.1745	3.6258	3.3345	1.1422	1.0504	8.03%

**Table 5:** BO-LSTM’S optimal hyperparameters of the modified models.

Set No.	*η*	*h*	*q*	*p*	*s*	*b*	*λ*	Iterations
1	4.2 × 10^−2^	123	1	0.40	30	16	1.0 × 10^−6^	15,000
2	4.8 × 10^−2^	50	1	0.26	53	30	6.4 × 10^−4^	23,000

**Table 6:** BSs and improvement of conventional and modified models.

Set No.	*BS*_7_ (dps)	*BS*_8_ (10^−2^ dps)	*BS*_9_ (10^−2^ dps)	*Q*_5_ (10^−2^)	*Q*_6_ (10^−2^)	|*Q*_6_ − *Q*_5_|/*Q*_5_
1	3.1877	3.9089	3.3135	1.2263	1.0395	15.23%
2	3.1824	4.2556	3.5623	1.3372	1.1194	16.29%

**Table 7:** BSs and improvement of conventional and modified models of I3G4250D in verifications.

Set No.	Axis	*BS*_10_ (dps)	*BS*_11_ (10^−2^ dps)	*BS*_12_ (10^−2^ dps)	*Q*_7_ (10^−2^)	*Q*_8_ (10^−2^)	|*Q*_8_ − *Q*_7_|/*Q*_7_
1	x	2.2245	2.7481	2.2861	1.2354	1.0277	16.81%
	y	2.9895	2.5012	2.0967	0.8367	0.7014	16.17%
	z	0.8585	2.0149	1.5226	2.3470	1.7736	24.43%
2	x	2.2307	2.8575	2.2849	1.2810	1.0243	20.04%
	y	2.9970	3.0015	2.3632	1.0015	0.7885	21.27%
	z	0.8575	2.1359	1.5883	2.4908	1.8522	25.64%

## Data Availability

The original contributions presented in this study are included in the article. Further inquiries can be directed to the corresponding author.

## References

[1] Abbasi J., Hashemi M., Alasty A. (2022). A Memory-Based Filter for Long-Term Error De-Noising of MEMS-Gyros. IEEE Trans. Instrum. Meas..

[2] Xi J.Q., Zheng Z.Y., Liu H.F., Zhang P. (2024). Towards True In-situ Temperature Compensation Utilizing Multiple Parameter Decoupling for Resonant MEMS Sensors Subject to Blue Sideband Excitation. IEEE Sens. Lett..

[3] Zhao Z.M., Yang L.H., Sun Q., Li F.B., Shen B.X., Ye A. (2024). Residual Error Integral Predictor-Based Smith Fuzzy PID Temperature Controller for Thermal Vacuum Test. IEEE Trans. Instrum. Meas..

[4] Wu X.Y., Wang X.N. A Fuzzy Self-tuning Temperature PID Control Algorithms for 3D Bio-printing Temperature Control System. Proceedings of the 2020 32nd Chinese Control and Decision Conference.

[5] Dong H., Zhang W., Li M.X., Zeng Z.B., Cao D., Li X.P. (2022). High-precision air temperature control considering both hardware elements and controller design. Case Stud. Therm. Eng..

[6] Krysko V.A., Awrejcewicz J., Yakovleva T.V., Kirichenko A.V., Szymanowska O., Krysko V.A. (2019). Mathematical modeling of MEMS elements subjected to external forces, temperature and noise, taking account of coupling of temperature and deformation fields as well as a nonhomogenous material structure. Commun. Nonlinear Sci. Numer. Simul..

[7] Maj C., Szermer M., Zajac P., Amrozik P. Analytical modelling of MEMS Z-axis comb-drive accelerometer. Proceedings of the 2019 20th International Conference on Thermal, Mechanical and Multi-Physics Simulation and Experiments in Microelectronics and Microsystems (EuroSimE).

[8] Vatanparvar D., Shkel A.M. (2022). On Correlation of Anisoelasticity, Angular Gain, and Temperature in Whole-Angle CVGs. IEEE Sens. J..

[9] Ma L., Chen W.W., Li B., Chen Z.G., You Z. Thermal modeling and compensation of MEMS accelerometer. Proceedings of the 3rd Asian Pacific Conference on Mechanical Components and Control Engineering.

[10] Ruzza G., Guerriero L., Revellino P., Guadagno F.M. (2018). Thermal Compensation of Low-Cost MEMS Accelerometers for Tilt Measurements. Sensors.

[11] Günhan Y., Unsal D. Polynomial Degree Determination for Temperature Dependent Error Compensation of Inertial Sensors. Proceedings of the 2014 IEEE/ION Position, Location and Navigation Symposium.

[12] Gheorghe M. (2017). Advanced Calibration Method with Thermal Compensation for 3-Axis MEMS Accelerometers. Rom. J. Inf. Sci. Technol..

[13] Ma C.Y., Lin J., Zhao Y., Shi Q., Xia G.M., Qiu A.P., Huang J.Y. Low Noise Temperature Compensation Strategy for North-finding MEMS Gyroscope. Proceedings of the 2024 IEEE Sensors.

[14] Kim B., Hopcroft M.A., Candler R.N., Jha C.M., Agarwal M., Melamud R., Chandorkar S.A., Yama G., Kenny T.W. (2008). Temperature Dependence of Quality Factor in MEMS Resonators. J. Microelectromech. Syst..

[15] Bekkeng J.K. (2009). Calibration of a Novel MEMS Inertial Reference Unit. IEEE Trans. Instrum. Meas..

[16] Qi B., Shi S.S., Zhao L., Cheng J.H. (2022). A Novel Temperature Drift Error Precise Estimation Model for MEMS Accelerometers Using Microstructure Thermal Analysis. Micromachines.

[17] Liu Z.M., Ayazi F. (2023). A Review of Eigenmode and Frequency Control in Piezoelectric MEMS Resonators. IEEE Trans. Ultrason. Ferroelectr. Freq. Control.

[18] Wu T.H., Zhang J., Gu M., Jiang J.L., Li Z., Lin C., Su Y. (2023). Analysis and Verification of the Temperature Drift Characteristics of MEMS Resonant Sensors During Power-On Startup. IEEE Trans. Instrum. Meas..

[19] Li C., Xing C.D., Wu J., Zhou M., Qiao W. (2025). Error Mechanisms and Calibration Scheme for Low Bias Mode-Matched Micro Resonant Gyroscopes Under Ocean Environment. IEEE Trans. Instrum. Meas..

[20] Basarab M., Giani A., Combette P. (2020). Thermal Accelerometer Simulation by the R Functions Method. Appl. Sci..

[21] Cheng J., Fang J., Wu W., Li J. (2014). Temperature drift modeling and compensation of RLG based on PSO tuning SVM. Measurement.

[22] Li K., Cui R., Cai Q., Wei W.Q., Shen C., Tang J., Shi Y.B., Cao H.L., Liu J. (2024). A Fusion Algorithm for Real-Time Temperature Compensation and Noise Suppression With a Double U-Beam Vibration Ring Gyroscope. IEEE Sens. J..

[23] Pan Y.J., Li L.L., Ren C.H., Luo H.L. (2010). Study on the compensation for a quartz accelerometer based on a wavelet neural network. Meas. Sci. Technol..

[24] Ma T.C., Cao H.L., Shen C. (2020). A Temperature Error Parallel Processing Model for MEMS Gyroscope Based on a Novel Fusion Algorithm. Electronics.

[25] Xu D., Yang Z., Zhao H., Zhou X. A temperature compensation method for MEMS accelerometer based on LM-BP neural network. Proceedings of the 2016 IEEE Sensors.

[26] Wang C.C., Lin C.J. (2023). Dynamic Analysis and Machine Learning Prediction of a Nonuniform Slot Air Bearing System. ASME J. Comput. Nonlinear Dyn..

[27] Wang S.D., Zhu W.L., Shen Y.J., Ren J., Gu H.R., Wei X.Y. (2020). Temperature compensation for MEMS resonant accelerometer based on genetic algorithm optimized backpropagation neural network. Sens. Actuators A-Phys..

[28] Wang C.C., Kuo P.H., Chen G.Y. (2022). Machine Learning Prediction of Turning Precision Using Optimized XGBoost Model. Appl. Sci..

[29] Cheng J.H., Qi B., Chen D.D., Landry R.J. (2015). Modification of an RBF ANN-Based Temperature Compensation Model of Interferometric Fiber Optical Gyroscopes. Sensors.

[30] Li Z., Cui Y.C., Gu Y.K., Wang G.D., Yang J., Chen K., Cao H.L. (2023). Temperature Drift Compensation for Four-Mass Vibration MEMS Gyroscope Based on EMD and Hybrid Filtering Fusion Method. Micromachines.

[31] Wang X.W., Cao H.L. (2022). Improved VMD-ELM Algorithm for MEMS Gyroscope of Temperature Compensation Model Based on CNN-LSTM and PSO-SVM. Micromachines.

[32] Qi B., Cheng J.H., Wang Z.L., Jiang C., Jia C. (2024). A Novel Temperature Drift Error Estimation Model for Capacitive MEMS Gyros Using Thermal Stress Deformation Analysis. Micromachines.

[33] Yariv E. (2022). Edge corrections for parallel-plate capacitors. Eur. J. Appl. Math..

[34] Wintle H.J., Kurylowicz S. (1985). Edge Corrections for Strip and Disc Capacitors. IEEE Trans. Instrum. Meas..

[35] Gao M., Bie X.R., Wang Y., Li Y.H., Zhai Z.Y., Lyu H.Q., Zou X.D. (2025). Accurate Deep Potential Model of Temperature-Dependent Elastic Constants for Phosphorus-Doped Silicon. Nanomaterials.

[36] Hochreiter S., Schmidhuber J. (1997). Long Short-Term Memory. Neural Comput..

[37] Mockus J. On Bayesian methods for seeking the extremum. Proceedings of the Optimization Techniques IFIP Technical Conference.

[38] Snoek J., Larochelle H., Adams R.P. Practical Bayesian optimization of machine learning algorithms. Proceedings of the Advances in Neural Information Processing Systems (NIPS) 25.

